# Fibroblast Activation Protein Expression in Sarcomas

**DOI:** 10.1155/2023/2480493

**Published:** 2023-06-09

**Authors:** Jacquelyn N. Crane, Danielle S. Graham, Christine E. Mona, Scott D. Nelson, Alireza Samiei, David W. Dawson, Sarah M. Dry, Marwan G. Masri, Joseph G. Crompton, Matthias R. Benz, Johannes Czernin, Fritz C. Eilber, Thomas G. Graeber, Jeremie Calais, Noah C. Federman

**Affiliations:** ^1^Department of Pediatrics, Division of Pediatric Hematology, Oncology, Stem Cell Transplantation & Regenerative Medicine, Stanford University School of Medicine, 1000 Welch Rd, Suite 300, Palo Alto, CA 94304, USA; ^2^University of California Los Angeles, Department of Surgery, Los Angeles, CA, USA; ^3^University of California Los Angeles, Department of Molecular and Medical Pharmacology, Los Angeles, CA, USA; ^4^University of California Los Angeles, Department of Pathology and Laboratory Medicine, Los Angeles, CA, USA; ^5^University of California Los Angeles, Jonsson Comprehensive Cancer Center, Los Angeles, CA, USA; ^6^University of California Los Angeles, Department of Pediatrics, Los Angeles, CA, USA

## Abstract

**Objectives:**

Fibroblast activation protein alpha (FAP) is highly expressed by cancer-associated fibroblasts in multiple epithelial cancers. The aim of this study was to characterize FAP expression in sarcomas to explore its potential utility as a diagnostic and therapeutic target and prognostic biomarker in sarcomas.

**Methods:**

Available tissue samples from patients with bone or soft tissue tumors were identified at the University of California, Los Angeles. FAP expression was evaluated via immunohistochemistry (IHC) in tumor samples (*n* = 63), adjacent normal tissues (*n* = 30), and positive controls (*n* = 2) using semiquantitative systems for intensity (0 = negative; 1 = weak; 2 = moderate; and 3 = strong) and density (none, <25%, 25–75%; >75%) in stromal and tumor/nonstromal cells and using a qualitative overall score (not detected, low, medium, and high). Additionally, RNA sequencing data in publicly available databases were utilized to compare FAP expression in samples (*n* = 10,626) from various cancer types and evaluate the association between FAP expression and overall survival (OS) in sarcoma (*n* = 168).

**Results:**

The majority of tumor samples had FAP IHC intensity scores ≥2 and density scores ≥25% for stromal cells (77.7%) and tumor cells (50.7%). All desmoid fibromatosis, myxofibrosarcoma, solitary fibrous tumor, and undifferentiated pleomorphic sarcoma samples had medium or high FAP overall scores. Sarcomas were among cancer types with the highest mean FAP expression by RNA sequencing. There was no significant difference in OS in patients with sarcoma with low versus high FAP expression.

**Conclusion:**

The majority of the sarcoma samples showed FAP expression by both stromal and tumor/nonstromal cells. Further investigation of FAP as a potential diagnostic and therapeutic target in sarcomas is warranted.

## 1. Introduction

Sarcomas are a heterogenous family of malignancies arising from bone, cartilage, or connective tissues [1]. There are approximately 16,000 new cases of sarcoma and 6,500 deaths from sarcoma each year in the United States [2]. Despite multimodal treatment, which may include surgery, radiation, and/or chemotherapy, five-year overall survival (OS) for patients with sarcomas is approximately 60 percent [3–5].

Given the heterogeneity and poor prognosis of sarcomas, there is a great interest in identifying novel diagnostic and therapeutic targets. Fibroblast activation protein alpha (FAP) has been proposed as a potential target in various cancer types [6–14]. FAP is a type II membrane-bound glycoprotein enzyme with protease activity [15]. FAP has been shown to be highly expressed by cancer-associated fibroblasts (CAFs) in multiple epithelial cancers with generally absent FAP expression in the tumor cells [7, 16–18]. Additionally, in epithelial cancers, FAP expression has been found to be associated with proliferation, angiogenesis, migration, invasion, and poor prognosis [8, 19–26]. Prior studies have shown that the majority of normal adult tissues have low or absent FAP expression [7, 8, 27, 28]. However, increased FAP expression has been reported in pancreatic islet alpha cells, endometrial cells, multipotent bone marrow stromal cells, fetal mesenchymal tissues, and pathologic sites of fibroblast activation, such as wound healing or inflammation [7, 15, 27–33]. Thus, FAP expression is associated with activated proliferating mesenchymal cells in fetal development, healing, and malignancy [7, 15, 28–31, 34].

FAP is an important marker of CAFs. CAFs are heterogenous cells which are key components of tumor stroma or the tumor microenvironment [7, 35]. Various phenotypic and functional subtypes have been described including myofibroblastic, inflammatory, antigen-presenting, and vascular CAFs although there is not currently a consensus classification system or nomenclature for CAF subtypes [6, 36, 37]. As a group, CAFs shape the extracellular matrix (ECM) and contribute to metabolic and immune reprogramming [8, 35]. CAFs have been shown to promote cancer cell survival, growth, metastasis, chemoresistance, and immune evasion [35, 38–41].

Given the differential expression of FAP, with limited to absent expression in normal tissues and high expression in CAFs, approaches to diagnostically and therapeutically target FAP as a way to indirectly target cancer cells are of great interest. As sarcomas are derived from mesenchymal cells, FAP may also be used as a direct target for cancer cells in sarcomas. However, the literature on FAP expression in sarcomas is limited [25, 27, 42]. Thus, a more comprehensive understanding of FAP expression in sarcomas is critical to understand its potential utility as an imaging target for diagnosis, staging, treatment response evaluation, and as a potential therapeutic target. This includes understanding expression of FAP by CAFs and tumor cells in the sarcoma microenviroment.

Herein, our first aim was to analyze the expression of FAP via immunohistochemistry (IHC) in tissue samples of patients with bone and soft tissue tumor subtypes, including sarcomas, available at our center. Our second aim was to analyze the expression of FAP by RNA sequencing and its association with OS using publicly available data.

## 2. Methods

### 2.1. Sample Selection

We conducted an institutional review board approved (IRB #10–001857) retrospective chart review of pathology reports of patients with a diagnosis of a bone or soft tissue tumor made between January 1, 2007 to July 31, 2020 and pathology sample available at the University of California, Los Angeles. We identified 73 available tumor samples from 59 patients with a pathologically confirmed bone or soft tissue tumor diagnosis. Samples were collected from various treatment time points including both pre- and postchemotherapy and/or radiation and were collected from both primary and metastatic sites. Additionally, we identified 37 available adjacent normal tissue samples collected at the same time as the tumor biopsies or resections in 36 patients. All samples were re-reviewed by a pathologist (S.N.) with expertise in sarcoma to select optimal representative tissue blocks from tumor and adjacent normal tissues.

### 2.2. Immunohistochemistry

**Figure 1:**
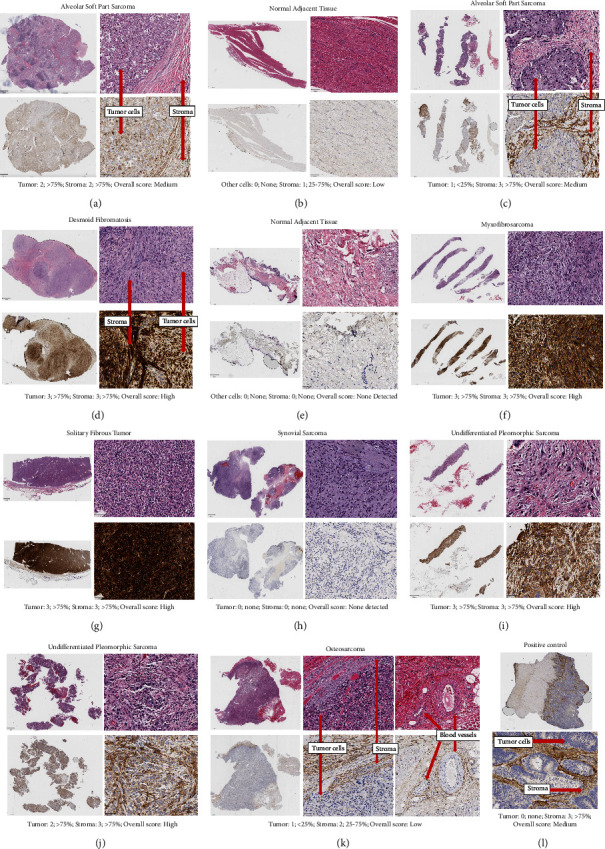
Representative H&E and FAP IHC images. In panels a–j, top images show low and high power views of H&E slide and bottom images show low and high power views of FAP stained slide. In panel (k), top left shows low power view of H&E slide, top middle shows high power view of H&E slide, bottom left shows low power view of FAP stained slide, bottom middle shows high power view of FAP stained slide, and top and bottom right shows high power view of FAP stained slide with arrows pointing to blood vessels. In panel (l), top image shows low power view of FAP stained slide and bottom image shows high power view of FAP stained slide. Scale bars are shown for each image. (a) A case of ASPS (FAP.ASPS.2.primary) with FAP staining in both tumor and stromal cells; (b) normal tissue adjacent to the ASPS case shown in panel A (FAP.ASPS.2.normal) with low intensity FAP staining in some stromal cells; (c) a case of ASPS (FAP.ASPS.3.primary) with FAP staining most notable in stromal cells; (d) a case of DF (FAP.DF.3.primary) with intense and dense FAP staining; (e) normal adjacent tissue to the DF case shown in panel D without FAP staining; (f) a case of myxofibrosarcoma (FAP.MFS.4.primary.1) with intense and dense FAP staining seen in the majority of the sample; (g) SFT case (FAP.SFT.5.primary) with intense and dense FAP staining seen in the majority of the cells; (h) a case of SS (FAP.SS.1.recurrence) without FAP staining; (i) a case of UPS (FAP.UPS.4.primary) with intense and dense FAP staining seen in the majority of the sample; (j) a case of UPS (FAP.UPS.1.primary) with dense FAP staining seen in the majority of the sample with higher intensity in stromal cells compared to tumor cells; (k) a case of osteosarcoma (FAP.OS.4.primary.2) with FAP staining that is most notable in stromal cells sections around blood vessels; and (l) colon cancer positive control.

Archival formalin-fixed paraffin-embedded tissue specimens were obtained from tumor biopsies or resections. Staining for hematoxylin and eosin (H&E) and FAP were performed on the specimens at the UCLA Tissue Pathology Core Laboratory. Polyclonal rabbit anti-FAP alpha antibody from Abcam was utilized (catalog number ab207178). Immunostaining was performed on Leica Bond platform. A positive control (colon cancer tumor sample) was used. All slides were scanned at 20X magnification. Samples were reviewed using the Orbit Image Analysis software and Figure 1 was made using the QuPath software [43, 44]. Seventeen of 110 samples (15%; *n* = 10 tumor; *n* = 7 normal) were excluded due to poor cellularity. Thus, 63 tumor samples from 53 patients and 30 adjacent normal tissue samples from 29 subjects were available for FAP IHC expression analysis. Assessment of IHC staining of each sample was performed by an oncologist (J.N.C.) and a pathologist (A.S.) and the consensus was reached. FAP staining intensity and density were graded semiquantitatively on a scale of 0–3 (0 = negative; 1 = weak; 2 = moderate; 3 = strong) and from 0-–100% (none, <25%, 25–75%; >75%), respectively. Additionally, a qualitative overall FAP expression score was assigned to each sample as follows: not detected, low, medium, and high.

For tumor samples, the stromal cells and tumor cells were scored separately. For adjacent normal samples, the stromal and nonstromal cells were scored separately. The average FAP intensity score was calculated for each of these cell types. The percentage of tumor and normal samples with positive FAP staining was calculated. FAP positive staining was defined as FAP IHC intensity score of ≥2 and FAP IHC density score ≥25%.

### 2.3. RNA Sequencing Data Acquisition, Processing, and Analysis

Publicly available RNA sequencing data from patient tumor samples were acquired from (1) The Cancer Genome Atlas (TCGA) via the University of California Santa Cruz Computational Genomics Lab Xena Browser (data available at http://xena.ucsc.edu/ under datahub TCGA Pan-Cancer); (2) the Therapeutically Applicable Research to Generate Effective Treatments (TARGET) osteosarcoma dataset (data available from https://portal.gdc.cancer.gov/projects with dbGaP study accession number phs000218); and (3) from the lab of Alejandro Sweet-Cordero (Sayles et al. manuscript; data available at https://ega-archive.org/under Dataset ID EGAS00001003201) [45, 46]. The TCGA data from Xena Browser was downloaded in toil processed form and the remainder of the data was processed through the toil pipeline in our laboratory. Toil is a computational pipeline through which raw RNA sequencing files can be processed in a uniform manner in order to minimize batch effects in conducting meta-analyses of RNA sequencing data from different experimental runs or datasets [47]. Upper quartile normalized counts were generated from the toil processed data files. All data were log transformed (log2(*x* + 1)) and FAP expression was compared across cancer subtypes. OS data for patients with sarcomas was downloaded from Xena Browser. Quality of the OS data from the TCGA has been previously reported [48]. Survival analysis was performed using the survival package in R. OS was compared between subjects with FAP expression in the upper and lower quartiles and *p* value was calculated based on chi-square testing.

## 3. Results

### 3.1. Sample Selection

A total of 63 tumor samples, 30 samples from normal tissue adjacent to the tumor samples, and 2 positive controls (colon cancer tumor samples) had adequate cellularity for review of H&E slides and quantification of FAP staining. A summary of tumor types and sample sources is shown in Table 1.

Further details about each sample, including treatment status, are included in Supplemental File 1. Of the 63 tumor samples, the tumor subtypes included were angiosarcoma (*n* = 2; 3.2%), alveolar soft part sarcoma (ASPS) (*n* = 4; 6.4%), desmoid fibromatosis (DF) (*n* = 5; 7.9%), Ewing sarcoma (*n* = 7; 11.1%), leiomyosarcoma (*n* = 7; 11.1%), well differentiated liposarcoma (*n* = 4; 6.3%), dedifferentiated liposarcoma (*n* = 4; 6.4%), pleomorphic liposarcoma (*n* = 1; 1.6%), myxofibrosarcoma (MFS) (*n* = 5; 7.9%), osteosarcoma (*n* = 5; 7.9%), solitary fibrous tumor (SFT) (*n* = 4; 6.4%), synovial sarcoma (SS) (*n* = 6; 9.5%), and undifferentiated pleomorphic sarcoma (UPS) (*n* = 9; 14.3%).

### 3.2. IHC Analysis

**Figure 2:**
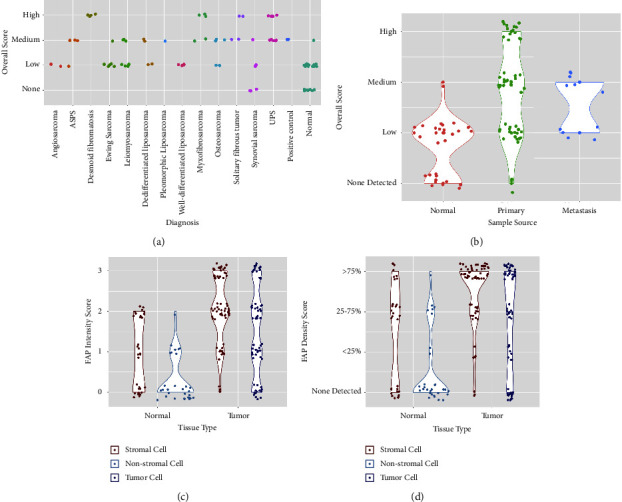
FAP scores by diagnosis and sample type. In each subfigure, a dot represents an individual sample; dots are offset from center to enable visualization of each individual sample. (a) FAP overall scores (none detected, low, medium, and high) are shown by diagnosis. (b-c) FAP scores are shown by sample type (normal tissue adjacent to tumor, primary tumor, and metastatic tumor) with FAP overall scores shown in panel b; FAP intensity scores (0, 1, 2, and 3) shown in panel c; and FAP density scores (none detected, <25%, 25-75%, and >75%) shown in panel d.

Representative H&E and FAP stained images are shown in Figure 1, including tumor and normal samples with varying FAP intensity, density, and overall scores. This includes a case of ASPS with normal adjacent tissue (panels A and B, respectively), an additional case of ASPS (panel C), a case of DF with normal adjacent tissue (panels D and E, respectively), a case of MFS (panel F), a case of SFT (panel G), a case of SS (panel H), two cases of UPS (panels I and L), a case of osteosarcoma (panel K), and the positive control. Summaries of FAP scores are shown in Figure 2 and Table 2. The details of the FAP IHC expression results for individual samples are listed in Supplemental File 1.

As shown in Table 2, the majority of tumor samples had positive FAP staining of stromal cells (77.7%) and tumor cells (50.7%). Conversely, of the normal samples, only 36.7% had positive FAP staining of stromal cells (36.7%) and none (0%) had positive FAP staining of nonstromal cells.

As shown in Figure 2, all DF (*n* = 5), MFS (*n* = 5), SFT (*n* = 4), and UPS (*n* = 9) samples had overall scores of medium or high. As shown in supplemental file 1, in all DF samples, the tumor and stromal cell FAP intensity scores were 3 and FAP density scores were >75%. Additionally, in all samples of MFS, SFT, and UPS, both tumor and stromal cell FAP intensity scores were ≥2 and FAP density scores were ≥25% with the exception of 1 UPS sample with a tumor FAP intensity score of 1. Of note, FAP staining was observed around blood vessels and representative images are shown in Figure 1(k).

### 3.3. RNA Sequencing

**Figure 3:**
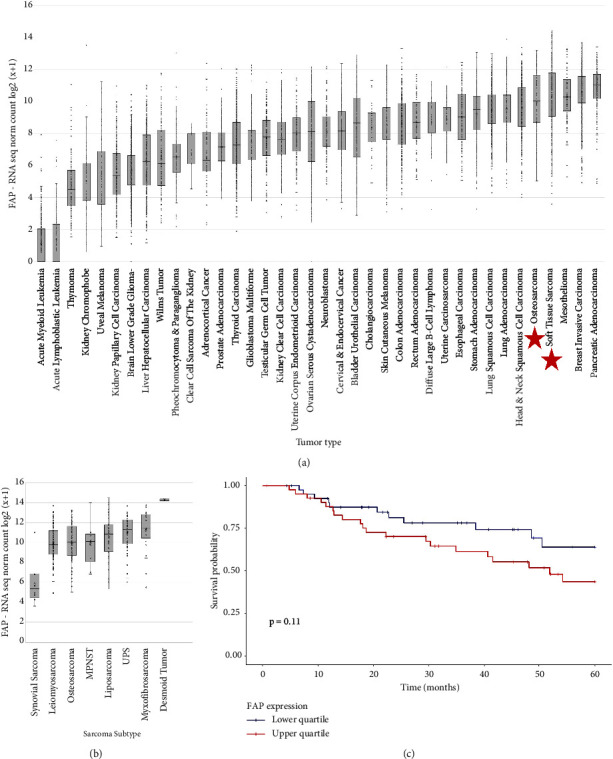
FAP gene expression by RNA sequencing from TCGA, TARGET, and Sayles et al. (a) FAP gene expression across multiple cancer types. Log transformed upper quartile normalized counts (log2(*x* + 1)) are shown. The tumor types were ranked by mean expression and then displayed in order from lowest to highest mean expression from left to right. (b) FAP gene expression by RNA sequencing across sarcoma subtypes. (c) Kaplan–Meier plot of OS based on FAP expression for subjects for whom both toil processed RNA sequencing data and survival data are available. OS is shown for those with FAP expression in the upper (*n* = 42) and lower quartiles (*n* = 42).

RNA sequencing data from 10,626 tissue samples from different cancer types were analyzed and shown in Figure 3(a). Osteosarcoma (*n* = 96) and soft tissue sarcoma (*n* = 262) were among cancer types with the highest FAP expression. RNA sequencing data from the 358 sarcoma samples (10 SS, 106 leiomyosarcoma, 57 liposarcoma, 25 MFS, 52 UPS, 10 malignant peripheral nerve sheath tumor (MPNST), 2 DF, and 96 osteosarcoma samples) are shown in Figure 3(b). The highest level of FAP RNA expression was observed in DF and the lowest in SS.

OS and RNA sequencing data were available for 168 patients with sarcoma (9 SS, 45 leiomyosarcoma, 27 liposarcoma, 13 MFS, 29 UPS, 5 MPSNT, 2 DF, and 38 osteosarcoma). As shown in Figure 3(c), OS was not significantly different between the patients with sarcoma with low FAP expression (lower quartile Q1, *n* = 42) and the patients with sarcoma with high FAP expression (upper quartile Q4, *n* = 42) (*p* = 0.11). Given the diversity of clinical behavior of sarcoma subtypes, survival analysis was also performed for individual sarcoma subtypes with the largest numbers of patients (leiomyosarcoma, UPS, osteosarcoma), and again was not significantly different between the patients with FAP expression in the lower quartile compared to the upper quartile.

## 4. Discussion

Prior studies have shown that FAP is highly expressed by CAFs with limited to no expression in normal adult tissues [7, 8, 16–18, 27, 28]. This, coupled with the cancer supportive role of CAFs, makes FAP a promising diagnostic and therapeutic target. The majority of the existing literature is focused on FAP expression in epithelial cancers and there is limited data on FAP expression in sarcomas. The data in sarcomas include studies by Rettig et al., Dohi et al., and Yuan et al. [25, 27, 42]. The current study adds to this work through the inclusion of additional bone and soft tissue tumor subtypes by IHC, tumors from both primary and metastatic sites, and various treatment time points.

In the current study, the expression of FAP by IHC and RNA sequencing was shown to be variable across the different bone and soft tissue tumor subtypes with the highest expression occurring in DF, MFS, SFT, and UPS samples. DF, MFS, and SFT are classified as fibroblastic and myofibroblastic tumors [49]. Thus, given the fibroblastic and myofibroblastic proliferations that characterize these tumors, it is not surprising that these tumor types show intense FAP expression. In epithelial cancers, FAP expression is largely limited to stromal cells although tumor cell expression of FAP has been reported in some epithelial cancer types including glioblastoma and uterine squamous cell carcinoma [8, 14]. In the current study, the majority of sarcoma samples showed FAP expression by both stromal and tumor cells.

Prior studies, including a meta-analysis which evaluated multiple cancer types including osteosarcoma, have found that high expression of FAP is associated with poor outcomes [22]. However, in the current study we did not find a significant difference in overall survival when comparing subjects with RNA sequencing FAP expression in the upper and lower quartiles. It must be noted that this survival analysis was performed in a small number of subjects in a limited number of sarcoma subtypes. Thus, while FAP expression was not found to be a prognostic biomarker in our analysis, future analyses may further clarify if it is prognostic in certain sarcoma subtypes.

Advances in the management of sarcomas, including those subtypes with the highest FAP expression levels, is needed. DF does not have metastatic potential and is considered a locally aggressive soft tissue tumor rather than a true sarcoma. Still, it can impact vital structures and result in morbidity and death [50]. MFS is associated with high risk of local relapse [51]. SFT is considered an intermediate malignancy which generally behaves in a benign and indolent manner although a subset of SFTs has the propensity for local recurrence and metastasis [52]. UPS is high-grade aggressive soft tissue sarcoma that lacks a specific line of differentiation [53].

Fluorodeoxyglucose (FDG) positron emission tomography (PET) is used frequently in the staging of sarcomas but has variable utility among the different sarcoma subtypes [54]. Thus, there is a need for improved imaging approaches for diagnosis, staging, and therapy response evaluation in sarcomas. Radiopharmaceuticals that target FAP, called FAP inhibitors (FAPI), have been recently developed. FAPI-PET tracers have shown promising results to date with high tumor-to-background contrast ratios in different cancer types [10–13, 55, 56].

Previous studies have shown promising results of FAPI-PET tracers in sarcoma. [10, 13, 57]. Specifically, a recent prospective observational trial including 47 patients with sarcoma imaged with 68-Gallium (^68^Ga)-FAPI-PET showed a significant association between FAPI-PET uptake intensity and FAP histopathologic expression as well as high sensitivity and predictive positive value of FAPI-PET [10]. Additionally, in 45 patients with recurrent soft tissue sarcoma, Gu and colleagues showed that ^68^Ga-DOTA-FAPI-04 PET detected more lesions and had improved sensitivity, specificity, positive predictive value, and negative predictive value compared to 18F-FDG PET.

Further investigation is needed to understand if FAPI PET tracers may provide increased sensitivity for tumor detection compared to other imaging approaches in sarcoma. Currently, there are multiple active clinical trials recruiting patients for FAP-targeted imaging for which patients with sarcoma may be eligible as shown in Table 3.

Given the overall poor prognosis of sarcomas, novel therapeutics are greatly needed. The cancer supportive functions of the tumor stroma and FAP expression by CAFs have stimulated the investigation of approaches to therapeutically target FAP in various cancer types [7, 37, 59, 60]. Given the high expression of FAP in both stromal and tumor cells in various sarcoma subtypes, further studies to therapeutically target FAP in sarcomas are warranted. The immunosuppressive effects of FAP and stromal cells further raise interest in immunotherapy approaches [39–41]. FAP is a potential target antigen for immunotherapeutic approaches including antibody-drug conjugates, bispecific T-cell engagers (BiTE), and chimeric antigen receptor (CAR)-T cell therapies [9].

Multiple preclinical studies of FAP-targeting immunotherapies in various cancer types have shown potential promise and limited toxicities [61–68]. However, of note, one study showed bone marrow toxicity and lethal cachexia in mice bearing pancreatic ductal adenocarcinomas treated with FAP-targeting CAR-Tcells [58]. With regards to sarcomas, in humanized NOD-*scid IL2Rγ*^*null*^ mice inoculated with human fibrosarcoma cells expressing FAP, the combination of anti-FAP-F19-ΔCD28/CD3*ζ*-CAR-T cells and PD-1 blockade demonstrated significant anti-tumor activity and improved survival [65].

Clinical studies of FAP-targeting therapeutics are also underway. A phase 1 clinical trial investigating sibrotuzumab, a humanized monoclonal antibody directed against FAP, in various cancers known to have FAP positivity had no objective tumor responses but it was tolerable [69]. Additionally, in a phase I clinical trial of F19 FAP-CAR-T-cells in patients with malignant pleural mesothelioma (NCT01722149), no evidence of treatment toxicity was observed and there was persistence of CAR-T cells in the peripheral blood [66, 70, 71]. Additionally, Kratochwil et al. recently reported disease control in several patients with sarcomas treated with FAP-targeted radioligand therapies [72, 73].  Table 4 shows ongoing therapeutic trials targeting FAP including for which patients with sarcoma may be eligible.

Further work to characterize FAP expression in normal tissue is needed to understand the potential for on-target off-tumor effects with FAP-targeted interventions. In the current study, FAP staining was seen around blood vessels confirming prior reports [8, 14]. FAP expression was also seen in a portion of the normal tumor-adjacent samples examined, though at lower levels than in the tumor samples, which has been reported previously [14]. It is possible that the normal tumor-adjacent tissue is influenced by the nearby tumor microenvionment. Therefore, we must be cautious in our interpretation of this data as the normal tissue samples obtained from specimen adjacent to the tumor may not be reflective of normal healthy tissue [74].

Limitations of the current study include limited number of sarcoma subtypes and total samples included. Further work is needed including investigation of FAP expression in other sarcoma subtypes and further evaluation of expression in normal tissues.

## 5. Conclusion

In this study, the expression of FAP by IHC and RNA sequencing was shown to be variable across the different bone and soft tissue tumor subtypes with the highest expression occurring in DF, MFS, SFT, and UPS samples. FAP may serve as a potential diagnostic and therapeutic target in certain sarcomas subtypes.

## Figures and Tables

**Table 1:** Immunohistochemistry case diagnoses and sample sources.

Diagnosis	Number of samples	Number of patients	Number of samples from primary site	Number of samples from metastatic site
Bone and soft tissue tumor subtypes	63	53	52	11
Angiosarcoma	2	2	2	0
Alveolar soft part sarcoma	4	4	3	1
Desmoid fibromatosis	5	4	5	0
Ewing sarcoma	7	3	5	2
Leiomyosarcoma	7	5	4	3
Dedifferentiated liposarcoma	4	4	4	0
Pleomorphic liposarcoma	1	1	1	0
Well differentiated liposarcoma	4	4	4	0
Myxofibrosarcoma	5	4	5	0
Osteosarcoma	5	4	2	3
Solitary fibrous tumor	4	4	4	0
Synovial sarcoma	6	6	5	1
Undifferentiated pleomorphic sarcoma	9	8	8	1
Adjacent normal	30	29	N/A	N/A
Positive control	2	1	1	0
Total	95	83	52	11

**Table 2:** FAP scores.

Sample type	Positive FAP staining	Average FAP intensity score	FAP overall score
Tumor samples	Stromal cells: 49 of 63 (77.7%) Tumor cells: 32 of 63 (50.7%)	Stromal cells: 2.0 Tumor cells: 1.5	(i) None detected in 3 of 63 (4.7%) (ii) Low in 23 of 63 (36.5%) (iii) Medium in 23 of 63 (36.5%) (iv) High in 14 of 63 (22.2%)

Normal samples	Stromal cells: 11 of 30 (36.7%) Nonstromal cells: 0 of 30 (0%)	Stromal cells: 0.96 Nonstromal cells: 0.3	(i) None in 11 of 30 (36.7%) (ii) Low in 18 of 30 (60%) (iii) Medium in 1 of 30 (3.3%) (iv) High in 0 (0%)

**Table 3:** Active clinical trials investigating PET imaging techniques targeting FAP for which patients with sarcoma may be eligible.

Intervention	Study population	Phase	NCT number
^68^Ga-FAPI-46 PET/CT	Adults with sarcoma scheduled to have biopsy or surgical resection	Phase 1	NCT04457258
^68^Ga-FAP-2286-PET	Adults with solid tumors	Phase 1	NCT04621435
^68^Ga-FAPI-46-PET/CT	Adults with malignancies	Phase 2	NCT05160051

Abbreviation key: FAP = fibroblast activation protein; F^68^Ga = 68-Gallium; API = fibroblast activation protein inhibitor; PET = positron emission tomography.

**Table 4:** Active clinical trials investigating therapeutics targeting FAP for which patients with sarcoma may be eligible.

Intervention	Intervention type	Study population	Phase	NCT number
177-Lu-FAP-2286	Peptide-targeted radionuclide therapy	Adults with refractory/progressive advanced/metastatic solid tumors	Phase 1/2	NCT04939610
MP0317	Designed ankyrin repeat protein (DARPin®) drug targeting FAP and CD40	Adults with relapsed/refractory advanced solid tumors	Phase 1	NCT05098405
RO7300490 as a single agent or combined with atezolizumab	RO7300490 is a bispecific antibody	Adults with advanced solid tumors	Phase 1	NCT04857138
RO6874281 as a single agent or combined with trastuzumab or cetuximab	RO6874281 is a bispecific targeted IL-2 variant with the antibody component targeted against FAP	Adults with advanced/metastatic solid tumors	Phase 1	NCT02627274
Nectin4/FAP targeted fourth-generation CAR-T cells	CAR-T	Adults with advanced malignant solid tumors	Phase 1	NCT03932565

Abbreviation key: FAP = fibroblast activation protein; CAR-T: chimeric antigen receptor (CAR)-T cell therapies.

## Data Availability

The results published here are in part based upon data generated by the Therapeutically Applicable Research to Generate Effective Treatments (TARGET) initiative (https://ocg.cancer.gov/programs/target) (data available at https://portal.gdc.cancer.gov/projects with dbGaP study accession number phs000218), The Cancer Genome Atlas (TCGA) (https://www.cancer.gov/tcga) processed by the University of California Santa Cruz (data available at http://xena.ucsc.edu/under datahub TCGA Pan-Cancer), and the lab of Dr. Alejandro Sweet-Cordero (Sayles et al. paper; data available at https://ega-archive.org/under Dataset ID EGAS00001003201) [45, 46]. Files of scanned IHC slides are available upon request from the corresponding author.

## Supplementary Materials

Supplementary Materials This file includes details about each sample including subject identifier, sample name, notes regarding relationship between tumor and adjacent normal samples, tumor diagnosis, FAP tumor/other cell intensity and density scores, FAP stromal cell intensity and density scores, FAP overall score, sample source (Primary tumor, metastasis, adjacent normal), and treatment status.
