# Supplementary material for: Fibroblast Activation Protein Expression in Sarcomas
Source: Sarcoma. 2023 Jun 9;2023:2480493. doi: 10.1155/2023/2480493 (PMC10275689; doi:10.1155/2023/2480493)
Supplement: Supplementary Materials — This file includes details about each sample including subject identifier, sample name, notes regarding relationship between tumor and adjacent normal samples, tumor diagnosis, FAP tumor/other cell intensity and density scores, FAP stromal cell intensity and density scores, FAP overall score, sample source (Primary tumor, metastasis, adjacent normal), and treatment status. [file 2480493.f1.docx]

| Supplemental Document 1. FAP IHC case details | | | | | | | | | | |  |
| --- | --- | --- | --- | --- | --- | --- | --- | --- | --- | --- | --- |
| Subject Name | **Sample Name** | **Note** | **Diagnosis** | **FAP tumor / other cell intensity score** | **FAP tumor / other cell density score** | **FAP stromal cell intensity score** | **FAP stromal cell density score** | **Composite score** | **Sample Source (Primary Tumor, Metastasis, Adjacent Normal)** | **Treatment status** | **Site** |
| Colon Cancer FAP positive control | Colon Cancer FAP positive control |  | Positive control | 0 | none | 3 | >75% | Medium | Primary | Unknown | Colon |
| Colon Cancer FAP positive control | Colon Cancer FAP positive control |  | Positive control | 0 | none | 3 | >75% | Medium | Primary | Unknown | Colon |
| FAP.Angiosarc.5 | FAP.Angiosarc.5.Primary |  | Angiosarcoma | 1 | >75% | 2 | >75% | Low | Primary | Treatment Naïve | Breast |
| FAP.Angiosarc.6 | FAP.Angiosarc.6.Primary |  | Angiosarcoma | 0 | none | 2 | <25% | Low | Primary | Treatment Naïve | Chest wall |
| FAP.ASPS.2 | FAP.ASPS.2.Primary |  | ASPS | 2 | >75% | 2 | >75% | Medium | Primary | Treatment Naïve | Thigh |
| FAP.ASPS.2 | FAP.ASPS.2.Normal | Normal Adjacent to FAP.ASPS.2.Primary | Normal | 0 | None | 1 | 25-75% | Low | Normal | N/A | Thigh |
| FAP.ASPS.3 | FAP.ASPS.3.Primary |  | ASPS | 1 | <25% | 3 | >75% | Medium | Primary | Treatment Naïve | Thigh |
| FAP.ASPS.4 | FAP.ASPS.4.Metastasis |  | ASPS | 2 | >75% | 2 | >75% | Medium | Metastasis | Treatment Naïve | Lung |
| FAP.ASPS.4 | FAP.ASPS.4.Normal | Normal Adjacent to FAP.ASPS.4.Metastasis | Normal | 1 | 25-75% | 2 | 25-75% | Low | Normal | N/A | Lung |
| FAP.ASPS.5 | FAP.ASPS.5.Primary.1 |  | ASPS | 1 | 25-75% | 1 | >75% | Low | Primary | Treatment Naïve | Extremity |
| FAP.DF.2 | FAP.DF.2.Primary |  | Desmoid fibromatosis | 3 | >75% | 3 | >75% | High | Primary | Treatment Naïve | Abdominal wall |
| FAP.DF.3 | FAP.DF.3.Primary |  | Desmoid fibromatosis | 3 | >75% | 3 | >75% | High | Primary | Treatment Naïve | Abdominal wall |
| FAP.DF.3 | FAP.DF.3.Recurrence |  | Desmoid fibromatosis | 3 | >75% | 3 | >75% | High | Primary, Recurrence | Treatment Naïve | Abdominal wall |
| FAP.DF.3 | FAP.DF.3.Normal.1 | Normal Adjacent to FAP.DF.3.Recurrence | Normal | 0 | None | 0 | None | None detected | Normal | N/A | Abdominal wall |
| FAP.DF.4 | FAP.DF.4.Primary |  | Desmoid fibromatosis | 3 | >75% | 3 | >75% | High | Primary | Treatment Naïve | Abdominal wall |
| FAP.DF.4 | FAP.DF.4.Normal | Normal Adjacent to FAP.DF.4.Primary | Normal | 1 | 25-75% | 2 | >75% | Medium | Normal | N/A | Abdominal wall |
| FAP.DF.5 | FAP.DF.5.Primary |  | Desmoid fibromatosis | 3 | >75% | 3 | >75% | High | Primary | Treatment Naïve | Abdominal wall |
| FAP.DF.5 | FAP.DF.5.Normal | Normal Adjacent to FAP.DF.5.Primary | Normal | 0 | None | 0 | None | None detected | Normal | N/A | Abdominal wall |
| FAP.EWS.1 | FAP.EWS.1.Primary.1 |  | Ewings Sarcoma | 0 | None | 3 | 25-75% | Low | Primary | Treatment Naïve | Thigh |
| FAP.EWS.1 | FAP.EWS.1.Primary.2 |  | Ewings Sarcoma | 0 | None | 2 | 25-75% | Low | Primary | Post neo-adjuvant chemotherapy | Thigh |
| FAP.EWS.1 | FAP.EWS.1.Normal | Normal Adjacent to FAP.EWS.1.Primary.2 | Normal | 2 | <25% | 2 | >75% | Low | Normal | N/A | Extremity |
| FAP.EWS.1 | FAP.EWS.1.Metastasis |  | Ewings Sarcoma | 0 | None | 2 | >75% | Low | Metastasis | Post chemotherapy | Lung |
| FAP.EWS.2 | FAP.EWS.2.Primary.1 |  | Ewings Sarcoma | 0 | None | 2 | >75% | Medium | Primary | Treatment Naïve | Thigh |
| FAP.EWS.2 | FAP.EWS.2.Primary.2 |  | Ewings Sarcoma | 1 | <25% | 1 | >75% | Low | Primary | Post neo-adjuvant chemotherapy | Thigh |
| FAP.EWS.2 | FAP.EWS.2.Normal | Normal Adjacent to FAP.EWS.2.Primary.2 | Normal | 1 | 25-75% | 2 | >75% | Low | Normal | N/A | Extremity |
| FAP.EWS.3 | FAP.EWS.3.Primary |  | Ewings Sarcoma | 0 | None | 1 | 25-75% | Low | Primary, Recurrence | Post chemotherapy, recurrence | Humerus |
| FAP.EWS.3 | FAP.EWS.3.Normal | Normal Adjacent to FAP.EWS.3.Primary | Normal | 1 | 25-75% | 2 | >75% | Low | Normal | N/A | Extremity |
| FAP.EWS.3 | FAP.EWS.3.Metastasis |  | Ewings Sarcoma | 1 | <25% | 2 | >75% | Low | Metastasis, Recurrence | Post chemotherapy, recurrence | Lung |
| FAP.Leiomyo.2 | FAP.Leiomyo.2.Primary |  | Leiomyosarcoma | 1 | 25-75% | 2 | >75% | Medium | Primary | Treatment Naïve | Retroperitoneum |
| FAP.Leiomyo.2 | FAP.Leiomyo.2.Normal | Normal Adjacent to FAP.Leiomyo.2.Primary | Normal | 0 | None | 2 | 25-75% | Low | Normal | N/A | Retroperitoneum |
| FAP.Leiomyo.4 | FAP.Leiomyo.4.Primary |  | Leiomyosarcoma | 2 | >75% | 2 | >75% | Medium | Primary | Post neo-adjuvant chemotherapy | Abdominal wall |
| FAP.Leiomyo.4 | FAP.Leiomyo.4.Normal | Normal Adjacent to FAP.Leiomyo.4.Primary | Normal | 1 | >75% | 2 | >75% | Low | Normal | N/A | Abdominal wall |
| FAP.Leiomyo.5 | FAP.Leiomyo.5.Primary |  | Leiomyosarcoma | 2 | >75% | 2 | >75% | Medium | Primary | Treatment Naïve | Abdomen |
| FAP.Leiomyo.7 | FAP.Leiomyo.7.Metastasis |  | Leiomyosarcoma | 1 | <25% | 1 | >75% | Low | Metastasis | Post chemotherapy in the distant past | Pelvis |
| FAP.Leiomyo.7 | FAP.Leiomyo.7.Recurrence |  | Leiomyosarcoma | 1 | >75% | 1 | >75% | Low | Primary, Recurrence | Post chemotherapy, recurrence | Abdomen/pelvis |
| FAP.Leiomyo.8 | FAP.Leiomyo.8.Metastasis.1 |  | Leiomyosarcoma | 1 | >75% | 1 | >75% | Low | Metastasis | Treatment Naïve | Gluteal |
| FAP.Leiomyo.8 | FAP.Leiomyo.8.Normal | Normal Adjacent to FAP.Leiomyo.8.Metastasis.1 | Normal | 0 | None | 0 | None | None detected | NA | N/A | Gluteal |
| FAP.Leiomyo.8 | FAP.Leiomyo.8.Metastasis.2 |  | Leiomyosarcoma | 1 | <25% | 1 | <25% | Low | Metastasis | Treatment Naïve | Extremity |
| FAP.Leiomyo.8 | FAP.Leiomyo.8.Normal.2 | Normal Adjacent to FAP.Leiomyo.8.Metastasis.2 | Normal | 0 | None | 0 | None | None detected | NA | N/A | Extremity |
| FAP.Lioposarc.1 | FAP.Lioposarc.1.Primary |  | Pleomorphic Liposarcoma | 2 | 25-75% | 2 | >75% | Medium | Primary | Post neo-adjuvant XRT | Pubis/groin |
| FAP.Lioposarc.1 | FAP.Lioposarc.1.Normal | Normal Adjacent to FAP.Lioposarc.1.Primary | Normal | 0 | None | 1 | 25-75% | Low | Normal | N/A | Pubis/groin |
| FAP.Lioposarc.3 | FAP.Lioposarc.3.Primary |  | Liposarcoma - dedifferentiated | 0 | None | 1 | <25% | Low | Primary | Treatment Naïve | Omentum |
| FAP.Lioposarc.4 | FAP.Lioposarc.4.Primary |  | Liposarcoma - dedifferentiated | 1 | 25-75% | 2 | 25-75% | Low | Primary, Recurrence | Post-chemotherapy and XRT in distant past, recurrence | Abdominal wall |
| FAP.Liposarc.5 | FAP.Liposarc.5.Primary |  | Liposarcoma - dedifferentiated | 2 | >75% | 2 | >75% | Medium | Primary | Treatment Naïve | Retroperitoneum |
| FAP.Liposarc.6 | FAP.Liposarc.6.Primary |  | Liposarcoma - dedifferentiated | 2 | >75% | 2 | >75% | Medium | Primary | Treatment Naïve | Retroperitoneum |
| FAP.Liposarc.6 | FAP.Liposarc.6.Normal | Normal Adjacent to FAP.Liposarc.6.Normal | Normal | 0 | None | 1 | 25-75% | Low | Normal | N/A | Retroperitoneum |
| FAP.WDLPS.1 | FAP.WDLPS.1.Primary |  | Liposarcoma - well differentiated | 1 | <25% | 1 | 25-75% | Low | Primary | Treatment Naïve | Thigh |
| FAP.WDLPS.2 | FAP.WDLPS.2.Primary |  | Liposarcoma - well differentiated | 1 | <25% | 2 | >75% | Low | Primary | Treatment Naïve | Thigh |
| FAP.WDLPS.3 | FAP.WDLPS.3.Primary |  | Liposarcoma - well differentiated | 1 | 25-75% | 2 | 25-75% | Low | Primary | Treatment Naïve | Retroperitoneum |
| FAP.WDLPS.4 | FAP.WDLPS.4.Primary |  | Liposarcoma - well differentiated | 1 | 25-75% | 2 | 25-75% | Low | Primary, Recurrence | Post chemotherapy, recurrence | Retroperitoneum |
| FAP.MFS.1 | FAP.MFS.1.Recurrence |  | Myxofibrosarcoma | 3 | >75% | 3 | >75% | High | Primary Recurrence | Post XRT, recurrence | Extremity |
| FAP.MFS.2 | FAP.MFS.2.Primary |  | Myxofibrosarcoma | 2 | 25-75% | 2 | 25-75% | Medium | Primary | Treatment Naïve | Extremity |
| FAP.MFS.2 | FAP.MFS.2.Normal | Normal Adjacent to FAP.MFS.2.Primary | Normal | 0 | None | 1 | 25-75% | Low | Normal | N/A | Extremity |
| FAP.MFS.3 | FAP.MFS.3.Primary |  | Myxofibrosarcoma | 3 | >75% | 3 | >75% | High | Primary | Treatment Naïve | Hip |
| FAP.MFS.3 | FAP.MFS.3.Normal | Normal Adjacent to FAP.MFS.3.Primary | Normal | 0 | None | 1 | 25-75% | Low | Normal | N/A | Hip |
| FAP.MFS.4 | FAP.MFS.4.Primary.1 | There are 2 slides from this sample | Myxofibrosarcoma | 3 | >75% | 3 | >75% | High | Primary | Treatment Naïve | Extremity |
| FAP.MFS.4 | FAP.MFS.4.Primary.1 | There are 2 slides from this sample | Myxofibrosarcoma | 3 | 25-75% | 3 | 25-75% | Medium | Primary | Treatment Naïve | Extremity |
| FAP.OS.3 | FAP.OS.3.Primary.1 |  | Osteosarcoma | 0 | None | 1 | <25% | Low | Primary | Treatment Naïve | Femur |
| FAP.OS.3 | FAP.OS.3.Metastasis |  | Osteosarcoma | 2 | >75% | 2 | >75% | Medium | Metastasis | Post-chemotherapy | Lung |
| FAP.OS.3 | FAP.OS.3.Normal.2 | Normal Adjacent to FAP.OS.3.Metastasis | Normal | 1 | 25-75% | 1 | 25-75% | Low | Normal | N/A | Lung |
| FAP.OS.4 | FAP.OS.4.Primary.2 |  | Osteosarcoma | 1 | <25% | 2 | 25-75% | Low | Primary | Post chemotherapy | Humerus |
| FAP.OS.5 | FAP.OS.5.Metastasis |  | Osteosarcoma | 2 | >75% | 2 | >75% | Medium | Metastasis, Recurrence | Post chemotherapy, recurrence | Lung |
| FAP.OS.6 | FAP.OS.6.Metastasis |  | Osteosarcoma | 2 | 25-75% | 3 | >75% | Medium | Metastasis, Recurrence | Post chemotherapy, recurrence | Lung |
| FAP.OS.1 | FAP.OS.1.Normal | Normal Adjacent to Osteosacoma, tumor sample not available | Normal | 0 | None | 0 | None | None detected | Normal | N/A | Extremity |
| FAP.OS.2 | FAP.OS.2.Normal | Normal Adjacent to Osteosacoma, tumor sample not available | Normal | 0 | None | 0 | None | None detected | Normal | N/A | Extremity |
| FAP.SFT.1 | FAP.SFT.1.Primary |  | Solitary fibrous tumor | 2 | 25-75% | 2 | 25-75% | Medium | Primary | Post XRT | Pelvis |
| FAP.SFT.1 | FAP.SFT.1.Normal | Normal Adjacent to FAP.SFT.1.Primary | Normal | 0 | None | 0 | None | None detected | Normal | NA | Pelvis |
| FAP.SFT.2 | FAP.SFT.2.Primary |  | Solitary fibrous tumor | 3 | >75% | 3 | >75% | High | Primary | Treatment Naïve | Pelvis |
| FAP.SFT.2 | FAP.SFT.2.Normal | Normal Adjacent to FAP.SFT.2.Primary | Normal | 1 | <25% | 0 | None | Low | Normal | NA | Pelvis |
| FAP.SFT.3 | FAP.SFT.3.Primary |  | Solitary fibrous tumor | 3 | 25-75% | 2 | 25-75% | Medium | Primary | Treatment Naïve | Breast |
| FAP.SFT.5 | FAP.SFT.5.Primary |  | Solitary fibrous tumor | 3 | >75% | 3 | >75% | High | Primary | Treatment Naïve | Pelvis |
| FAP.SS.1 | FAP.SS.1.Recurrence |  | Synovial sarcoma | 0 | None | 0 | None | None detected | Primary, Recurrence | Post neoadjuvant chemotherpay and XRT, recurrence | Extremity |
| FAP.SS.2 | FAP.SS.2.Metastasis |  | Synovial sarcoma | 0 | None | 3 | >75% | Medium | Metastasis | Post neoadjuvant chemotherpay and XRT | Extremity |
| FAP.SS.2 | FAP.SS.2.Normal | Normal Adjacent to FAP.SS.2.Metastasis | Normal | 0 | None | 2 | >75% | Low | Normal | NA | Extremity |
| FAP.SS.3 | FAP.SS.3.Primary |  | Synovial sarcoma | 0 | None | 0 | None | None detected | Primary | None | Extremity |
| FAP.SS.4 | FAP.SS.4.Primary |  | Synovial sarcoma | 0 | None | 0 | None | None detected | Primary | Post neoadjuvant chemotherpay and XRT | Extremity |
| FAP.SS.4 | FAP.SS.4.Normal.1 | Normal Adjacent to FAP.SS.4.Primary | Normal | 0 | None | 0 | None | None detected | Normal | NA | Extremity |
| FAP.SS.5 | FAP.SS.5.Primary |  | Synovial sarcoma | 0 | None | 1 | 25-75% | Low | Primary | Treatment Naïve | Extremity |
| FAP.SS.6 | FAP.SS.6.Primary |  | Synovial sarcoma | 0 | None | 1 | >75% | Low | Primary | Treatment Naïve | Extremity |
| FAP.UPS.1 | FAP.UPS.1.Primary |  | UPS | 2 | >75% | 3 | >75% | High | Primary | Treatment Naïve | Thigh |
| FAP.UPS.3 | FAP.UPS.3.Primary |  | UPS | 3 | >75% | 3 | >75% | High | Primary | Treatment Naïve | Thigh |
| FAP.UPS.3 | FAP.UPS.3.Normal | Normal Adjacent to FAP.UPS.3.Primary | Normal | 0 | None | 1 | 25-75% | Low | Normal | N/A | Thigh |
| FAP.UPS.4 | FAP.UPS.4.Primary |  | UPS | 3 | >75% | 3 | >75% | High | Primary | Treatment Naïve | Back |
| FAP.UPS.5 | FAP.UPS.5.Primary |  | UPS | 3 | >75% | 3 | >75% | High | Primary, Recurrence | Post chemotherapy in the distant past, recurrence | Thigh |
| FAP.UPS.5 | FAP.UPS.5.Normal | Normal Adjacent to FAP.UPS.5.Primary | Normal | 0 | None | 2 | 25-75% | Low | Normal | N/A | Thigh |
| FAP.UPS.6 | FAP.UPS.6.Primary |  | UPS | 2 | >75% | 2 | >75% | Medium | Primary | Treatment Naïve | Gluteal |
| FAP.UPS.6 | FAP.UPS.6.Normal | Normal Adjacent to FAP.UPS.6.Primary | Normal | 0 | None | 2 | 25-75% | Low | Normal | N/A | Gluteal |
| FAP.UPS.7 | FAP.UPS.7.Primary |  | UPS | 1 | 25-75% | 3 | >75% | Medium | Primary | Treatment Naïve | Shoulder |
| FAP.UPS.7 | FAP.UPS.7.Normal | Normal Adjacent to FAP.UPS.7.Metastasis | Normal | 0 | None | 2 | >75% | Low | Normal | N/A | Lung |
| FAP.UPS.7 | FAP.UPS.7.Metastasis |  | UPS | 2 | 25-75% | 2 | 25-75% | Medium | Metastasis | Treatment Naïve | Lung |
| FAP.UPS.9 | FAP.UPS.9.Primary |  | UPS | 2 | >75% | 2 | >75% | Medium | Primary | Treatment Naïve | Gluteal |
| FAP.UPS.9 | FAP.UPS.9.Normal | Normal Adjacent to FAP.UPS.9.Primary | Normal | 0 | None | 0 | None | None detected | Normal | N/A | Gluteal |
| FAP.UPS.10 | FAP.UPS.10.Primary |  | UPS | 2 | >75% | 2 | >75% | Medium | Primary | Treatment Naïve | Extremity |
| FAP.UPS.10 | FAP.UPS.10.Normal | Normal Adjacent to FAP.UPS.10.Primary | Normal | 0 | None | 0 | None | None detected | Normal | N/A | Extremity |
| FAP.UPS.11 | FAP.UPS.11.Normal | Normal Adjacent to UPS, tumor sample not available | Normal | 0 | None | 0 | None | None detected | Normal | N/A | Abdomen |
